# Multi-Strain Probiotic Intervention Modestly Modulates Microbial Composition and Inflammatory Profile in Individuals with Long COVID

**DOI:** 10.3390/microorganisms14040734

**Published:** 2026-03-25

**Authors:** Ana Bačić, Tijana Gmizić, Marija Branković, Mirjana Rajilić-Stojanović

**Affiliations:** 1 Innovation Centre of Faculty of Technology and Metallurgy, University of Belgrade, 11000 Belgrade, Serbia; abacic@tmf.bg.ac.rs; 2 Department of Gastroenterology and Hepatology, Clinic for Internal Medicine, University Hospital Medical Center “Bežanijska Kosa”, 11000 Belgrade, Serbiamanive23@gmail.com (M.B.); 3 Faculty of Medicine, University of Belgrade, 11000 Belgrade, Serbia; 4 Department of Biochemical Engineering and Biotechnology, Faculty of Technology and Metallurgy, University of Belgrade, 11000 Belgrade, Serbia

**Keywords:** gut microbiota, probiotics, long COVID, inflammation

## Abstract

Probiotics are widely used to support host health by modulating microbial communities and immune–metabolic homeostasis. Such interventions may be particularly relevant in long COVID syndrome, a condition characterized by persistent symptoms, low-grade inflammation, and microbiota alterations following SARS-CoV-2 infection. This study investigated the effects of a multi-strain probiotic on gut microbiota composition and predicted functional potential and biochemical parameters in individuals with long COVID and convalescent participants. Healthy individuals were included as reference controls. In an interventional study, 34 participants received a 12-week probiotic formulation containing *Saccharomyces boulardii*, *Lacticaseibacillus rhamnosus* GG, and two *Lactiplantibacillus plantarum* strains, while 40 served as non-supplemented controls. Fecal microbiota, assessed using 16S rRNA sequencing, and biochemical markers were measured at baseline and post-intervention. Probiotic supplementation induced selective compositional changes without significantly altering overall microbial diversity. Effects were more pronounced in long COVID participants and included enrichment of bacteria associated with metabolic and immune regulation, including *Adlercreutzia*, *Coprococcus*, and *Eubacterium*. Functional prediction analysis identified a probiotic-responsive signature in long-COVID-affected individuals, characterized by enrichment of pathways related to energy metabolism and redox balance. These microbial changes were accompanied by a consistent trend toward reduced inflammatory and hepatic markers. Overall, probiotic intervention demonstrated microbiota-status-dependent potential in long COVID recovery.

## 1. Introduction

Modulation of the gut microbiota has emerged as a promising therapeutic approach in the management of infectious, inflammatory, and metabolic diseases [1,2,3]. Gut microorganisms play a central role in maintaining host homeostasis through regulation of immune responses, metabolic processes, intestinal barrier integrity, and systemic inflammation [4,5,6]. Detrimental disruption of microbial ecosystem balance, commonly referred to as dysbiosis, has been associated with chronic low-grade inflammation, metabolic dysfunction, and impaired intestinal integrity across a variety of diseases [7,8].

Probiotic interventions have demonstrated potential in restoring disrupted microbial communities, regulating dysbiosis, and supporting immune and metabolic functions [9,10,11]. Beyond competing for nutrients as transient colonizers, probiotic strains may influence resident microbes through their metabolic activity by supporting intestinal integrity and regulating immune and inflammatory signaling [9,11,12,13]. Due to these properties, probiotics are increasingly explored as adjunctive therapeutic strategies in conditions characterized by prolonged immune and metabolic disturbances [14,15].

In this context, infection with severe acute respiratory syndrome coronavirus 2 (SARS-CoV-2), the cause of coronavirus disease 2019 (COVID-19), represents a particularly relevant model. Beyond its primary respiratory manifestations [16], SARS-CoV-2 affects multiple organ systems, including the gastrointestinal tract and residing microbes [17,18,19]. Through direct interactions with intestinal epithelial cells and indirect systemic effects, SARS-CoV-2 induces pronounced pro-inflammatory and immunomodulatory responses, disrupts the intestinal barrier, alters metabolic pathways, and interferes with gut-distal organ signaling [18,20,21]. Collectively, these mechanisms create a permissive environment for sustained microbial dysbiosis, consistently reported in COVID-19 [17,22,23,24].

Beyond the acute infection, a substantial proportion (up to 30–50%) of COVID-affected individuals experience multisystemic symptoms, persisting for months or even years after initial infection [25,26,27]. More than 200 symptoms have been described, collectively referred to as long COVID or post-COVID syndrome [28,29,30,31,32]. Accumulating evidence indicates that long COVID is associated with sustained alterations in gut microbial communities [31,33,34,35]. Disrupted gut microbiota may, in turn, contribute to the persistence of long COVID symptoms [35,36,37,38]. Although partial microbial recovery may occur over time [39,40], distinct signatures have been reported in individuals with long COVID more than a year after infection [41,42,43].

Given the limited therapeutic options available for long COVID [44,45], interventions aimed at restoring gut microbiota homeostasis, including probiotic supplementation, represent a promising therapeutic approach for alleviating persistent symptoms [37,46,47,48]. Probiotic supplementation has shown potential to support microbial recovery following SARS-CoV-2 infection by promoting beneficial commensals and enhancing the production of health-promoting metabolites, including short-chain fatty acids [37,49]. Most interventional studies in COVID-19 and long COVID populations have focused on *Lactobacillus* and *Bifidobacterium* strains, reporting improvements in microbial composition, inflammatory markers, and symptom alleviation [46,47,50]. In contrast, data on *Saccharomyces boulardii*, a probiotic yeast with established barrier-protective, immunomodulatory, and anti-inflammatory properties [51,52], remain scarce in the context of long COVID. Given that probiotic effects are strain- and formulation-specific [11], multi-strain combinations may provide broader functional coverage than single-strain interventions in alleviating long COVID symptoms.

Therefore, the present study aimed to investigate the effects of a multi-strain probiotic intervention on gut microbiota composition, predicted microbial functional potential, and selected biochemical parameters in individuals with long COVID and convalescent participants, with healthy individuals included as non-intervention reference controls.

## 2. Materials and Methods

### 2.1. Study Design and Participants

This study was designed as a double-blind, single-center, double-arm interventional study, with a previously published cross-sectional analysis of the same cohort [41]. The study was conducted at the University Hospital Medical Center Bežanijska Kosa (Belgrade, Serbia) between January and June 2023.

A total of 90 adult participants (≥18 years) were recruited. Participants were categorized into three clinical groups (n = 30 per group) based on infection history and symptom persistence:

(1) Individuals with long COVID syndrome, defined as persistent symptoms lasting at least 3 months following confirmed SARS-CoV-2 infection;
(2) Fully recovered convalescent controls with documented history of SARS-CoV-2 infection but no persistent symptoms;
(3) Healthy controls without a history of symptomatic COVID-19.

SARS-CoV-2 infection was confirmed by polymerase chain reaction (PCR) or antigen testing. Among participants with long COVID syndrome, the duration of persistent symptoms at enrollment ranged from 3 to 27 months following the initial infection, with a median duration of 14 months. During the acute phase of SARS-CoV-2 infection, infected participants received antibiotics and probiotics according to the standard treatment protocol at the University Hospital Medical Center Bežanijska Kosa [53]. Exclusion criteria included active SARS-CoV-2 or other acute infection, antibiotic or probiotic use within 3 months prior to sample collection, malignant disease, and other uncontrolled chronic conditions with the potential to substantially affect microbiota composition.

The study was designed to evaluate the effects of probiotic supplementation on fecal microbiota composition and biochemical parameters, as well as associations between microbial and biochemical changes over time. Baseline microbial composition of this cohort has been previously characterized in a cross-sectional study [41]. The present study builds on these findings by evaluating longitudinal changes associated with probiotic supplementation. Probiotic supplementation was administered only to participants with a history of SARS-CoV-2 infection (long COVID and convalescent groups), while healthy individuals served as non-intervention reference controls. Due to non-random allocation and unequal distribution of participants, the study was analyzed as an interventional study, rather than a randomized placebo-controlled trial.

The study was approved by the University Hospital Medical Centre “Bežanijska kosa” Ethics Committee (approval number 2237/2), and written informed consent was obtained from all participants.

### 2.2. Probiotic Intervention

The probiotic formulation consisted of *Saccharomyces boulardii* DBVPG-6763, *Lacticaseibacillus rhamnosus* GG, *Lactiplantibacillus plantarum* LP6595, and *L. plantarum* HEAL9 (EnteroBiotik^®^ FORTE, Abela Pharm, Belgrade, Serbia), providing a daily dose of at least ≥8.5 × 10^9^ viable colony-forming units (CFUs).

The probiotic was stored according to the manufacturer’s instructions at room temperature in a dry environment until administration. It was packaged using flow-pack technology, ensuring stability and preservation of viable microorganisms [54]. As a commercially validated formulation was used, no additional viability testing was performed.

Participants in the intervention group received the probiotic orally for 12 weeks, while participants in the control group did not receive probiotic supplementation. Compliance was monitored by self-report. To minimize subjectivity and placebo-related bias of this double-blinded intervention, the study focused on objective outcome measures, including microbiota profiling and laboratory-based biochemical markers.

### 2.3. Sample Collection and Biochemical Analysis

Fasting blood and fecal samples were collected at baseline and after the 12-week intervention period. A total of 90 were initially enrolled. Of these, 16 participants were excluded due to unclear sample labeling (n = 9) or incomplete paired sampling when the second timepoint was not provided (n = 7). As the analysis was based on paired baseline and post-intervention samples, only participants with complete sample pairs were included.

The final dataset comprised 74 paired samples from 23 participants with long COVID, 26 convalescent controls, and 25 healthy controls. Of these, 34 participants (13 with long COVID and 21 convalescent) received probiotic supplementation, while 40 participants (10 with long COVID) served as non-supplemented controls (Table 1).

Blood samples were analyzed using Mindray BS-800 (Mindray, Shenzhen, China). Biochemical analysis included complete blood count, glucose, lipid profile, urea, creatinine, total and direct bilirubin, liver enzymes (AST, ALT, ALP, GGT), LDH, CK, electrolytes (sodium, potassium, chloride), total proteins, albumin, C-reactive protein (CRP), D-dimer, iron, total iron-binding capacity, and ferritin.

### 2.4. Fecal Microbiota Analysis

Microbial DNA was extracted from fecal samples using the QIAamp Fast DNA stool mini kit (Qiagen, Hilden, Germany) according to the manufacturer’s instructions, with the addition of a bead-beating step [55]. DNA integrity was assessed with agarose gel electrophoresis, and extracted DNA samples were stored at −20 °C until sequencing.

The V_3_–V_4_ hypervariable region of the bacterial 16S rRNA gene was amplified and sequenced using paired-end sequencing on the Illumina NovaSeq 600 platform, conducted at Novogene (Beijing, China). Primers 341F (CCTAYGGGRBGCASCAG) and 806R (GGACTACNNGGGTATCTAAT) were used. Paired-end FASTQ files were generated, and sequencing quality was assessed using FastQC prior to downstream bioinformatics processing [56]. Sequence processing was performed using Quantitative Insights Into Microbial Ecology 2 (QIIME-2, version 2025.7) [57]. Raw paired-end demultiplexed FASTQ sequences were processed using the Divisive Amplicon Denoising Algorithm 2 (DADA2) pipeline for quality filtering, denoising, chimera removal, and amplicon sequence variant (ASV) inference [58]. Phylogenetic trees were constructed using the q2-phylogeny plugin [58], while taxonomic classification was performed using a naïve Bayes classifier trained on the SILVA reference database (version 138.2) [59,60].

### 2.5. Statistical Analysis

Statistical analysis was performed in R (version 4.5.0) using the RStudio interface (version 2025.05.0). QIIME-2-generated objects were imported into R using qiime2R [61]. Downstream microbiota analysis was conducted using phyloseq, microbiome, microbiomeStat, lme4, and ggplot2 packages [62,63,64,65,66]. Prior to analysis, generated ASVs were filtered by removing singletons, taxa present in fewer than 10% of samples, and taxa with relative abundance below 0.01%. Filtered ASV counts were normalized using total sum scaling (TSS) to obtain relative abundances. Rarefaction was not applied, as compositional data analysis approaches were used [67]. For differential abundance testing, data were subsequently transformed using centered log-ratio (CLR) transformation.

Analyses focused on probiotic-treated versus non-supplemented participants, with additional subgroup analysis within the long COVID cohort. Statistical significance was defined as *p* < 0.05, while results with 0.05 < *p* < 0.1 were reported as trends, as done by others [68,69]. Differences between groups were evaluated using Fisher’s exact test for categorical variables, including sex, time since infection, and long COVID severity, and Mann–Whitney U or Kruskal–Wallis tests for continuous variables (age).

Alpha diversity was assessed using the Shannon and Observed species indices, and differences among groups were investigated using the microbiomeStat alpha diversity functions [66]. Beta diversity was evaluated using Bray–Curtis and weighted UniFrac distances and visualized with Principal coordinate analysis (PCoA) plots. Differences among groups were tested by permutational multivariate analysis of variance (PERMANOVA) with 999 permutations, and permutational analysis of multivariate dispersions (PERMDISP) [70].

Differential abundance analysis was performed using the Linear Model for Differential Abundance Analysis (LinDA) on CLR-transformed genus-level data. Pairwise differences in log-fold changes (LFC) between groups were assessed with *generate_taxa_change_test_pair()* function from the microbiomeStat package [71]. Selected COVID-associated taxa were additionally analyzed individually using Student’s *t*-test on changes in relative abundance (Δ, t1–t0) to assess differences between probiotic and control groups using a targeted approach. Functional potential of the gut microbiota was assessed using Phylogenetic Investigation of Communities by Reconstruction of Unobserved States 2 (PICRUSt2, version 2.4.2) [72]. Predicted KEGG Orthologs were summarized into MetaCyc metabolic pathways [73,74]. Changes in pathway abundances were evaluated using linear mixed-effects models, with time, disease group, probiotic status, and their interactions as fixed effects, and participant ID as a random intercept.

Biochemical parameters were evaluated by comparing within-group paired sample changes (Δ, t1–t0) using Wilcoxon signed-rank tests. Spearman correlation analysis was used to investigate associations between changes in CLR-transformed microbial genera and biochemical parameters.

## 3. Results

### 3.1. Participant Characteristics

This study assessed the impact of a multi-strain probiotic intervention on fecal microbiota composition, predicted microbial functional potential, and biochemical parameters in individuals with long COVID and convalescent participants, with healthy non-supplemented individuals included as reference controls.

Baseline demographic characteristics of the study cohort are summarized in Table 2. No statistically significant differences were observed in age or sex distribution between long COVID, convalescent individuals, and healthy control groups (*p* > 0.05 for all comparisons).

Across the entire cohort, participants receiving probiotic supplementation and non-supplemented controls were comparable in age and sex distribution (Table 3).

Within the long COVID subgroup, no significant differences were observed between probiotic-treated and non-supplemented participants in age, sex distribution, time since infection, or symptom severity (Table 4, *p* > 0.05 for all comparisons).

Analyses were performed on paired baseline and post-intervention samples, with gut microbiota composition profiled using 16S rRNA gene amplicon sequencing. After sequence processing and quality control, sequencing generated a median of 45,980 reads per sample, with a total of 7,355,659 high-quality sequences retained for downstream analysis. In total, 8998 ASVs were identified, representing 323 genera across 103 bacterial families.

### 3.2. Microbial Diversity

Overall microbial diversity was assessed using alpha- and beta-diversity metrics. Alpha diversity, evaluated with Shannon and Observed species indices, showed no statistically significant differences between baseline and post-intervention samples across study groups (*p* > 0.05 for all comparisons).

**Figure 1:**
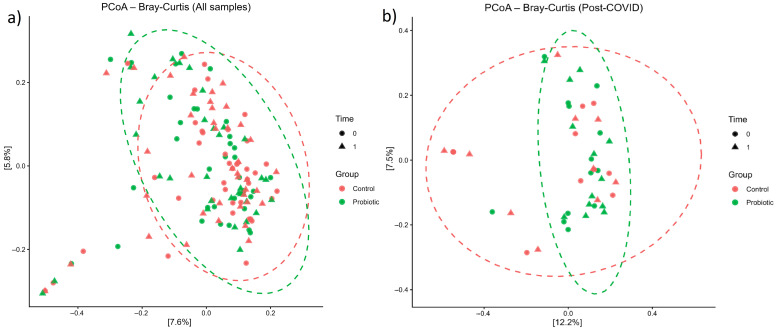
Principal Coordinates Analysis (PCoA) plots based on Bray–Curtis distances depicting gut microbial communities in probiotic and control groups at baseline (t0) and post-intervention (t1), including (**a**) all participants, and (**b**) long COVID subset.

Consistent with these findings, beta-diversity analyses revealed no significant changes in overall microbial community structure following probiotic supplementation. PERMANOVA based on Bray–Curtis distances showed no differences between probiotic and control groups (F = 0.83, *p* = 0.422), and dispersion did not differ significantly (F = 0.43, *p* = 0.51). Similar results were obtained for weighted UniFrac distances (*p* > 0.05). PcoA based on Bray–Curtis distances showed substantial overlap between probiotic and control samples, both in the full cohort and in the long COVID subgroup (Figure 1a,b).

### 3.3. Differential Microbiota Composition Analysis

Differential abundance analysis demonstrated that probiotic supplementation resulted in selective modulation of the gut microbial community compared with the control groups. The observed log-fold changes indicated a shift in microbial composition, with a subset of genera exhibiting significant alterations in abundance following the intervention, while the majority of taxa remained unchanged.

#### 3.3.1. Overall Microbiota Response to Probiotic Supplementation

**Figure 2:**
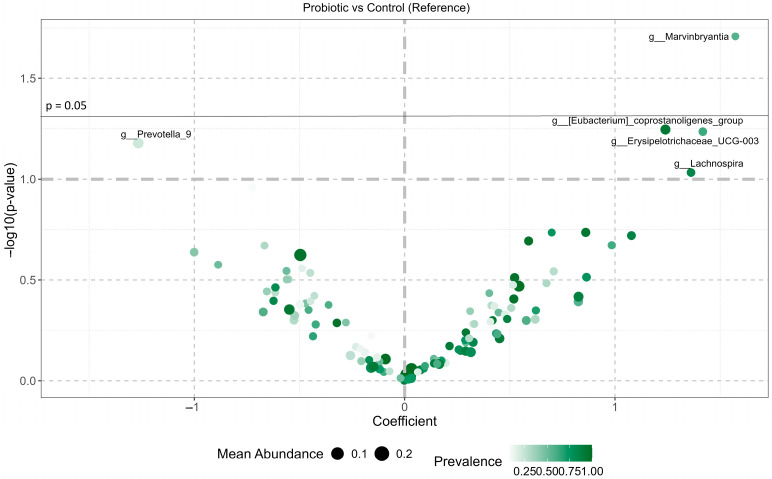
Volcano plot of paired genus-level abundance changes following probiotic intervention. Log-fold change coefficients and −log10(*p* values) are shown for genera abundance changes between baseline and post-intervention, comparing probiotic and control groups. Results were obtained using paired linear modeling. Positive coefficients indicate greater increases in the probiotic group relative to controls. Point size reflects the mean relative abundance, and color intensity indicates prevalence across samples.

Across the full cohort, probiotic supplementation was associated with modest but detectable changes in the abundance of several bacterial genera (Figure 2, Table S1). The most evident change was the increase in *Marvinbryantia* relative abundance (LFC = 1.57, *p* = 0.019). Additionally, a trend of increase following probiotic supplementation was observed for the *Eubacterium coprostanoligenes* group (*p* = 0.057), *Erysipelotrichaceae* UCG-003 (*p* = 0.058), and *Lachnospira* (*p* = 0.093). In contrast, a trend toward a decrease in *Prevotella*_9 abundance was detected in the probiotic group compared with controls (LFC = −1.27, *p* = 0.066).

#### 3.3.2. Long-COVID-Specific Response to Probiotic Supplementation

**Figure 3:**
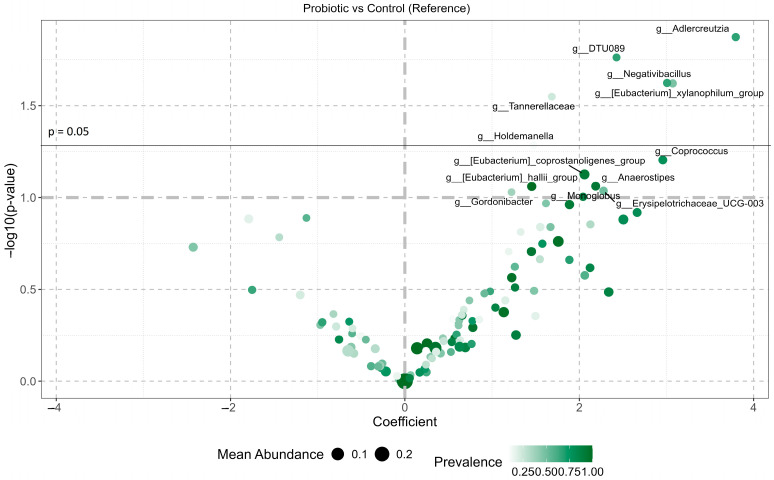
Volcano plot of differentially abundant genera in probiotic-treated participants with long COVID. Log-fold change coefficients and −log10(*p* values) are shown for genera abundance changes between baseline and post-intervention, comparing probiotic and control groups. Point size reflects the mean relative abundance, and color intensity indicates prevalence across samples.

The microbial response to probiotic intervention in long-COVID-affected participants was evident through significant changes in the abundance of five genera and 13 trend-level alterations across the three dominant phyla: Actinomycetota, Bacillota, and Bacteroidota (Figure 3, Table S2). Several genera were significantly enriched in the probiotic group compared with controls, including *Adlercreutzia* (*p* = 0.013), *Ruminococcaceae* DTU089 (*p* = 0.017), *Negativibacillus* (*p* = 0.024), the *Eubacterium xylanophilum* group (*p* = 0.024), and unclassified *Tannerellaceae* (*p* = 0.028). Additional genera exhibited trend-level increases in abundance (*p* < 0.1), including *Coprococcus*, the *Eubacterium coprostanoligenes* group, *Anaerostipes*, and the *Eubacterium hallii* group.

Overall, although probiotic supplementation was associated with modest compositional changes in the full cohort, a greater number of differentially abundant genera were observed in participants with long COVID.

#### 3.3.3. Changes in the Relative Abundance of Selected COVID-Associated Bacterial Genera

To complement the global differential abundance analysis, a targeted analysis of selected COVID-associated genera was performed. Focused analyses were performed to assess the effect of probiotic supplementation on the abundance of specific bacterial groups, which have been previously reported to be altered in COVID-19 and post-COVID conditions [17,75,76]. The change in the relative abundance of beneficial *Akkermansia*, *Bifidobacterium*, and *Faecalibacterium*, as well as potentially pathogenic *Eggerthella*, *Prevotella*, and *Prevotella_9*, was evaluated.

**Figure 4:**
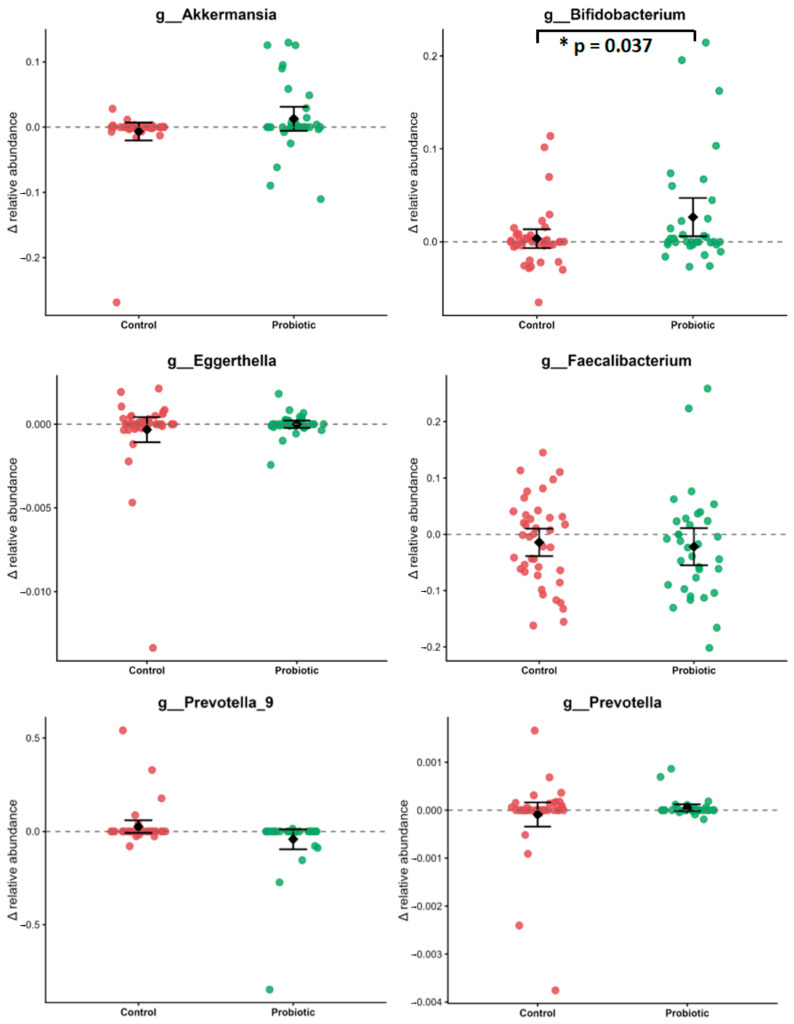
Change in relative abundance (Δ, t1–t0) of selected COVID-associated bacterial genera following probiotic intervention. Individual points represent participant-level changes in relative abundance for control and probiotic groups. Black points indicate group means, with error bars representing 95% confidence intervals. * indicates *p* < 0.05.

Following the intervention, differences in relative abundance changes between probiotic and control groups were observed for selected genera (Figure 4). A significant increase in *Bifidobacterium* was detected in the probiotic group compared with controls (Δ probiotic = 0.0264, Δ control = −0.0067, *p* = 0.0371). *Akkermansia* showed a trend toward higher abundance in the probiotic group (Δ probiotic = 0.0128, Δ control = 0.0034, *p* = 0.0829). Furthermore, a lower relative abundance of *Prevotella_9* was observed following probiotic supplementation compared with controls (Δ probiotic = 0.019, Δ control = −0.030, *p* = 0.013). No significant differences between groups were detected for *Faecalibacterium* (Δ probiotic = −0.0218, Δ control = −0.0144, *p* = 0.7089), *Eggerthella* (Δ probiotic = −2.2 × 10^−6^, Δ control = −3.21 × 10^−4^, *p* = 0.44), and *Prevotella* (Δ probiotic = −5.16 × 10^−5^, Δ control = −7.9 × 10^−5^, *p* = 0.32).

### 3.4. Functional Prediction Analysis

Functional prediction analysis was performed to evaluate changes in gut microbial functional potential following probiotic supplementation.

In the overall analysis including all probiotic recipients (long COVID and fully recovered individuals), probiotic supplementation was associated with a consistent trend toward enrichment of microbial biosynthetic and metabolic pathways over time (*p* < 0.1; Table S3). These pathways were primarily related to amino acid biosynthesis (including L-tryptophan), vitamin metabolism (folate, pantothenate, thiamine), and nucleotide-associated metabolic processes. Although these changes reached trend-level significance, enrichment was observed consistently across multiple functionally related pathways.

To assess whether probiotic-associated functional changes differed specifically in long COVID participants, a three-way interaction linear model was applied. This analysis identified a set of functional pathways significantly enriched in long COVID individuals receiving probiotics (*p* < 0.05; Table 5). These pathways were predominantly related to central energy metabolism and redox processes, including multiple ubiquinone and ubiquinol biosynthesis pathways.

### 3.5. Biochemical Parameters Analysis

Biochemical parameters were evaluated to examine probiotic-associated changes compared with the control. At baseline, no significant differences in biochemical markers were observed between study groups.

Across all participants, probiotic supplementation was associated with a borderline reduction in CRP (r = −0.405, *p* = 0.058). When analyses were restricted to the long COVID subgroup, participants receiving probiotics exhibited consistent directional trends toward lower liver enzyme levels (ALT and AST) and CRP, alongside higher serum ferritin concentrations compared with controls (*p* < 0.1, Table 6). Although none of these differences reached statistical significance, effect size estimates indicated moderate effects across the evaluated biomarkers (|r| ≈ 0.43–0.46).

### 3.6. Gut Microbiota and Biochemical Parameters Association

Spearman correlation analysis was performed using changes (Δ) in CLR-transformed genus abundances and corresponding changes in biochemical parameter levels. Correlations with |ρ| > 0.3 and *p* < 0.05 were considered statistically significant.

**Figure 5:**
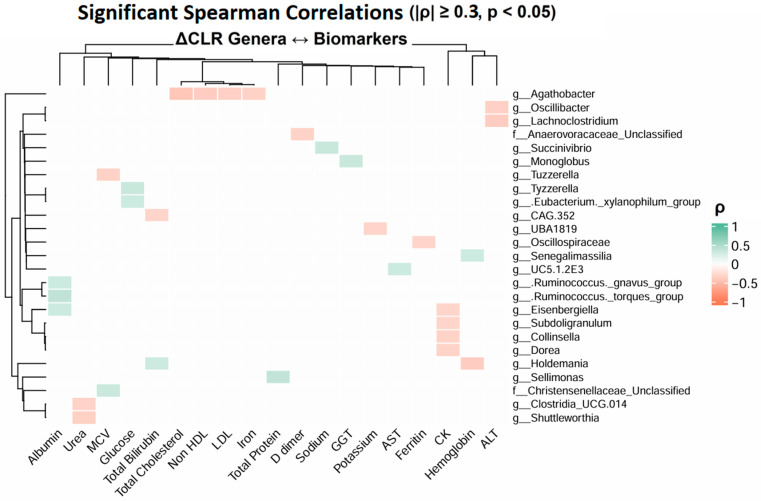
Clustered heatmap of Spearman correlation displaying Spearman correlation coefficients (ρ) between changes in CLR-transformed gut microbial genera and biochemical parameters in the overall cohort. ρ > 0.4, *p* < 0.05; Green indicates positive correlations, and red indicates negative correlations.

Across all participants, 30 significant associations between changes in microbial genera and biochemical parameters were identified (*p* < 0.05; Figure 5, Table S4), primarily involving markers of liver function and systemic inflammation. When the analysis was restricted to participants with long COVID, a total of 217 significant correlations were detected, indicating a stronger coupling between microbiota changes and host biochemical responses in long COVID-affected individuals. Several genera showed notable associations with liver enzymes. In particular, increases in *Negativibacillus*, *Eubacterium coprostanoligenes*, and *Collinsella* abundance were negatively correlated with ALT and AST levels. In addition, several bacterial groups, including *Tannerellaceae*, *Frisingicoccus*, *Gordonibacter*, and *Holdemanella*, showed significant associations with inflammatory and hepatic markers, including a negative association with CRP levels (*p* < 0.05, Figure S1, Table S5). In contrast, the abundance of *Prevotella_9* and *Intestinibacter* was positively correlated with CK levels, while *Collinsella*, *Coprobacter*, and *Erysipelotrichaceae* UCG-003 showed a negative correlation.

## 4. Discussion

Probiotic interventions are increasingly recognized as targeted strategies for supporting recovery of disrupted microbial ecosystems in conditions characterized by persistent dysbiosis and immune–metabolic imbalance [9,10,14,15]. Rather than inducing major shifts in microbiota structure, probiotics typically exert selective compositional and functional modulation of microbiota [9,12,13,77]. Such effects may be particularly relevant in incompletely recovered microbial communities, including those described in long COVID. Although previous probiotic studies in COVID-19 and long COVID have reported beneficial effects, study design, used strains, and outcomes vary considerably [37,46,47,48,49]. In our previous cross-sectional analysis of this cohort, significant differences in microbiota composition were observed in individuals with long COVID, whereas convalescent participants showed profiles comparable to healthy controls [41].

In the present interventional study, the effects of a multi-strain probiotic on gut microbiota composition, predicted functional potential, and biochemical parameters were evaluated in individuals with long COVID and convalescent participants, while healthy controls represented the reference group. Probiotic supplementation did not induce major shifts in overall microbial diversity or global community structure, as reflected by stable alpha- and beta-diversity metrics. This is consistent with the established understanding that probiotics primarily modulate specific taxa rather than altering global community diversity [12,78].

At the taxonomic level, probiotic intervention was responsible for modest but biologically relevant changes in bacterial abundance. These effects were more evident in the long COVID subgroup, where a greater number of differentially abundant taxa were observed following the intervention. This heightened responsiveness likely reflects persistent microbial and metabolic perturbations and greater ecological plasticity in long COVID, consistent with previous reports of sustained dysbiosis in this condition [42,43,79]. In contrast, the limited response observed at the overall cohort level suggests that individuals without persistent symptoms may have a more stable and less responsive microbial community, in line with evidence of microbial recovery following SARS-CoV-2 infection [80].

Differential abundance analysis revealed enrichment of genera associated with metabolic homeostasis and immune regulation in probiotic-treated long COVID participants, including *Adlercreutzia*, *Negativibacillus*, *Eubacterium*-related groups, and members of Ruminococcaceae. These taxa have previously been reported as depleted in acute COVID-19 and long COVID [42,81,82,83], suggesting that their increase may reflect partial microbial restoration. Many of these bacteria are involved in short-chain fatty acid production, immunomodulation, and regulation of inflammatory responses [84,85,86,87], processes potentially relevant to long COVID recovery. The enrichment of *Adlercreutzia* is particularly relevant given its role in polyphenol metabolism, anti-inflammatory, and other health-promoting properties [84,88]. Previous studies have also demonstrated that lactobacilli-containing probiotics can stimulate the growth of *Adlercreutzia* and *Coprococcus* [89], highlighting indirect effects of probiotics, mediated through microbial interactions.

Probiotic supplementation did not result in a generalized restoration of microbial taxa reported as altered in individuals with long COVID in our previous cross-sectional study. However, several probiotic-associated shifts were observed within specific bacterial lineages. Notably, multiple members of the Eggerthellaceae family have been previously identified as altered in association with long COVID status in this cohort and elsewhere [41,82,83]. Following probiotic intervention, compositional changes were observed within this group, including an increase in *Adlercreutzia*, although *Slackia* was decreased in the baseline. Both genera have been reported to possess potentially beneficial metabolic activities [84,88,90]. Although these changes do not indicate a complete normalization of the microbiota, they suggest that probiotic supplementation may influence specific microbial lineages associated with host metabolic functions.

To further assess the impact of probiotic intervention on key microbiota members, targeted analyses of selected COVID-associated genera were conducted. *Bifidobacterium* showed a significant increase, while *Akkermansia* demonstrated a trend toward enrichment following the intervention. Responses were heterogeneous, with substantial inter-individual variability, likely reflecting differences in baseline microbiota composition and host-related factors [91,92]. Furthermore, genera commonly enriched in inflammatory or dysbiotic states, like acute and long COVID conditions, including *Prevotella_9* and *Eggerthella* [46,81,93,94], were not stimulated by the intervention and, in some individuals, exhibited decreasing trends.

Functional prediction analysis further suggested potential disease-specific probiotic-associated functional shifts. Across all participants, trend-level enrichment of predicted biosynthetic pathways suggested modulation of microbial metabolic activity, including amino acid and vitamin metabolism pathways reported as altered in COVID conditions [81,95,96]. Importantly, a distinct and statistically significant probiotic-responsive functional signature was identified exclusively in long COVID participants. Predicted enriched pathways were predominantly related to microbial energy metabolism, including redox balance and quinone biosynthesis, indicating potential enhancement of microbial respiratory capacity and energetic efficiency. Given that functions have been found to be disrupted in long COVID [81,96], their predicted enrichment may reflect partial restoration of microbial activity.

Beyond microbiota modulation, probiotic supplementation was associated with modest yet directionally consistent biochemical changes. In long COVID participants receiving probiotics, trends toward reduced CRP and liver enzyme levels (ALT, AST), together with increased serum ferritin concentrations, were observed. Although not statistically significant, their consistency may suggest a potential attenuation of persistent systemic inflammation and hepatic biochemical alterations, recognized features of long COVID [37,97]. Correlation analysis further supported links between microbial shifts and host responses. Increases in *Negativibacillus* and the *Eubacterium coprostanoligenes* group were negatively correlated with ALT and AST concentrations, suggesting a potential relationship between enrichment of these taxa and improved hepatic biomarkers. Although causality cannot be inferred, these findings align with previous reports connecting gut microbiota composition with metabolic and inflammatory parameters in long COVID [98].

Previous probiotic studies in acute and long COVID populations have predominantly evaluated bacterial formulations containing *Lactobacillus* and *Bifidobacterium* strains, reporting variable outcomes [46,47,50,99,100,101]. In the present study, probiotic supplementation selectively enriched metabolically and immunologically relevant bacteria, with compositional changes most evident in long COVID participants. These findings provide preliminary evidence that multi-strain probiotic supplementation may modulate microbiota composition and functional potential in post-infection conditions.

Differences between studies are not unexpected and likely reflect formulation- and strain-specific effects, as well as microbiota baseline, host-, and geography-related variability [11,102,103]. Notably, our formulation included *S. boulardii*, a probiotic yeast not previously systematically evaluated in long COVID. Its inclusion may have contributed to the observed distinct compositional and functional responses, potentially through complementary mechanisms within the gut ecosystem. In addition to *S. boulardii*, other emerging probiotic candidates, including *Pediococcus* species, are increasingly being investigated for their role in microbial modulation and post-infectious recovery [104,105].

Despite the presence of *Lactobacillus* strains in the formulation, no increase in their relative abundance was detected. This observation is consistent with several previous studies indicating that probiotic effects may occur without stable colonization of the administered taxa [99,100].

This study has limitations, including its modest sample size, non-randomized design, and reliance on functional prediction inferred from 16S rRNA gene data rather than metagenomic measurements. In addition, as with all amplicon-based approaches, potential biases related to primer specificity and amplification efficiency should be considered when interpreting taxonomic profiles [106]. Given the sample size and variability inherent to microbiome data, several observations were identified as trends and should be interpreted cautiously.

Despite these limitations, the consistency of compositional, biochemical, correlation, and predicted functional findings supports a biologically coherent probiotic-associated modulation of the gut ecosystem, particularly in individuals with long COVID.

## 5. Conclusions

Multi-strain probiotic supplementation promoted the modulation of both taxonomic composition and predicted functional potential of the gut microbiota, with more evident effects in individuals with long COVID. Enrichment of selected beneficial taxa, together with a consistent trend of inflammatory and metabolic markers decrease, supports a potential role of probiotics in addressing post-infectious microbial and metabolic alterations. These findings suggest that targeted probiotic interventions may contribute to the restoration of gut ecosystem stability and host homeostasis during long COVID recovery. Confirmation of these observations in larger randomized controlled trials with strain-specific evaluation and multi-omics approaches is warranted.

## Figures and Tables

**Table 1:** Distribution of study participants by clinical groups and intervention status (probiotic vs. non-supplemented control).

Clinical Group	Probiotic (n)	Control (n)	Total (n)
Long COVID	13	10	23
Convalescent	21	5	26
Healthy controls	0	25	25
Total	34	40	74

**Table 2:** Baseline demographic characteristics of study participants across clinical groups. Differences between groups for sex distribution were evaluated using Fisher’s exact test and Kruskal–Wallis tests for continuous variables (age).

Characteristic	Long COVID (n = 23)	Convalescent (n = 26)	Healthy Controls (n = 25)	*p*-Value
Age (years), mean ± SD	45.3 ± 11.6	41.5 ± 8.9	43.9 ± 11.2	0.52
Female, n (%)	20 (87%)	21 (81%)	20 (80%)	0.75

**Table 3:** Baseline characteristics of participants according to intervention status. Differences between groups were evaluated using Fisher’s exact test for sex distribution and Mann–Whitney U tests for continuous variables (age).

Characteristic	Probiotic (n = 34)	Control (n = 40)	*p*-Value
Age (years), mean ± SD	43.5 ± 10.2	42.7 ± 10.8	0.77
Female, n (%)	30 (88%)	28 (70%)	0.09

**Table 4:** Baseline characteristics of long COVID participants according to probiotic intervention status. Differences between groups were evaluated using Fisher’s exact test for categorical variables and Mann–Whitney U tests for continuous variables (age).

Characteristic	Probiotic (n = 13)	Control (n = 10)	*p*-Value
Age (years), mean ± SD	47.2 ± 10.5	40.5 ± 9.9	0.15
Female, n (%)	12 (92%)	8 (80%)	0.56
Time since infection > 12 months, n (%)	9 (69%)	4 (40%)	0.23
Long COVID severity			
Mild, n (%)	3 (23%)	4 (40%)	0.74
Moderate, n (%)	5 (38%)	3 (30%)	
Severe, n (%)	4 (31%)	3 (30%)	

**Table 5:** MetaCyc metabolic pathways enriched following probiotic supplementation in individuals with long COVID. Effect estimates were obtained using linear model analysis to evaluate changes in pathway abundance between probiotic and control groups over time.

Pathway ID	MetaCyc Pathways	Effect Estimate	SE	T-Statistic	*p*-Value
PWY-6165	Chorismate biosynthesis II	0.691	0.266	2.598	0.011
P221-PWY	Octane oxidation	0.894	0.377	2.373	0.020
UBISYN-PWY	Superpathway of ubiquinol-8 biosynthesis (prokaryotes)	0.893	0.445	2.009	0.048
PWY-5855	Ubiquinol-7 biosynthesis	0.893	0.445	2.007	0.049
PWY-5856	Ubiquinol-8 biosynthesis (chorismate → 4-hydroxybenzoate)	0.893	0.445	2.007	0.049
PWY-5857	Ubiquinol-9 biosynthesis	0.893	0.445	2.007	0.049
PWY-6708	Ubiquinone-8 biosynthesis (prokaryotes)	0.893	0.445	2.007	0.049

**Table 6:** Changes in selected biochemical parameters following probiotic intervention compared with control. Median within-group changes (Δ, t1–t0) are shown for the probiotic and control groups in the overall cohort and within the long COVID subgroup. Differences between groups were assessed using the Wilcoxon signed-rank test applied to Δ values. Effect sizes are reported as rank correlation coefficients (r). *p* < 0.10 was considered indicative of a trend.

Variable	Probiotic Group (Median Δ)	Control Group (Median Δ)	*p*-Value	*r*
All participants				
CRP	−0.25	0	0.058	−0.405
Long COVID subgroup				
ALT	−0.5	2.5	0.073	−0.458
AST	−0.5	1.5	0.073	−0.458
CRP	−0.25	0.05	0.089	−0.433
Ferritin	14.5	0.5	0.099	0.425

## Data Availability

The raw sequence data have been deposited in the National Center for Biotechnology Information (NCBI) Sequence Read Archive (SRA) under the project number PRJNA1441616 and will be made publicly available upon acceptance of the manuscript.

## Supplementary Materials

The following supporting information can be downloaded at: https://www.mdpi.com/article/10.3390/microorganisms14040734/s1, Table S1: differentially abundant bacterial genera following probiotic supplementation compared with control. Log-fold change (LFC) coefficients were estimated using paired linear modeling of abundance changes over time. Positive LFC values indicate greater increases in abundance in the probiotic group relative to controls, whereas negative values indicate greater decreases. *p* < 0.05 was considered statistically significant, and *p* < 0.10 was considered a trend. SE, standard error. Table S2: differentially abundant bacterial genera in post-COVID participants following probiotic intervention compared with control. Log-fold change (LFC) coefficients were estimated from paired (t1–t0) changes in genus-level abundance using linear modeling. *p* < 0.05 was considered statistically significant, and *p* < 0.10 was considered a trend. SE, standard error. Table S3: MetaCyc metabolic pathways showing trend-level enrichment following probiotic supplementation in overall cohort. Functional pathway abundances were predicted from 16S rRNA gene data using PICRUSt2 and summarized at the MetaCyc level. Effect estimates were obtained using linear model analysis to evaluate changes in pathway abundance between probiotic and control groups over time. Table S4: Spearman correlation analysis results for long-COVID-affected individuals showing significant associations between changes (Δ, t1–t0) in CLR-transformed genus abundances and biochemical markers of liver function (ALT, AST) and systemic inflammation (CRP). Only correlations with |ρ| > 0.4 and *p* < 0.05 for selected biochemical markers are shown. Table S5. Spearman correlation analysis results for long COVID affected individuals showing significant associations between changes (Δ, t1–t0) in CLR-transformed genus abundances and biochemical markers of liver function (ALT, AST), systemic inflammation (CRP), D-dimer, ferritin, HDL, and non-HDL. Selected correlations with |ρ| > 0.3 and *p* < 0.05 for biochemical markers are shown. Figure S1. Clustered heatmap of Spearman correlation displaying Spearman correlation coefficients (ρ) between changes in CLR-transformed gut microbial genera and biochemical parameters in long COVID individuals. ρ > 0.5, *p* < 0.05; Green indicated positive correlations and red indicate negative correlations.

## Abbreviations

The following abbreviations are used in this manuscript:

SARS-CoV-2	Severe Acute Respiratory Syndrome Coronavirus 2
COVID-19	Coronavirus Disease 2019
CFU	Colony-forming Units
AST	Aspartate Aminotransferase
ALT	Alanine Aminotransferase
ALP	Alkaline Phosphatase
GGT	Gamma-Glutamyl Transferase
LDH	Lactate Dehydrogenase
CK	Creatine Kinase
CRP	C-reactive protein
QIIME-2	Quantitative Insights Into Microbial Ecology 2
DADA2	Divisive Amplicon Denoising Algorithm 2
ASV	Amplicon Sequence Variant
PCoA	Principal Coordinate Analysis
PERMANOVA	Permutational Multivariate Analysis of Variance
PERMDISP	Permutational Analysis of Multivariate Dispersions
LinDA	Linear Model for Differential Abundance Analysis
CLR	Centered log-ratio
PICRUSt2	Phylogenetic Investigation of Communities by Reconstruction of Unobserved States 2
KEGG	Kyoto Encyclopedia of Genes and Genomes
LFC	log-fold change
