# Supplementary material for: Multi-Strain Probiotic Intervention Modestly Modulates Microbial Composition and Inflammatory Profile in Individuals with Long COVID
Source: Microorganisms. 2026 Mar 25;14(4):734. doi: 10.3390/microorganisms14040734 (PMC13118416; doi:10.3390/microorganisms14040734)
Supplement: Supplementary file 1 [file microorganisms-14-00734-s001.zip › microorganisms-4201992-supplementary.pdf]

Table S1: Differentially abundant bacterial genera following probiotic supplementation compared with control. Log fold change (LFC) coefficients were estimated using paired linear modeling of abundance changes over time. Positive LFC values indicate greater increases in abundance in the probiotic group relative to controls, whereas negative values indicate greater decreases. p < 0.05 was considered statistically significant, and p < 0.10 was considered a trend. SE, standard error.

Genus	LFC Coefficient	SE	p value
Marvinbryantia	1.57	0.66	0.019
***Eubacterium coprostanoligenes* group**	1.24	0.64	0.057
***Erysipelotrichaceae* UCG-003**	1.42	0.74	0.058
Prevotella_9	-1.27	0.68	0.066
Lachnospira	1.36	0.80	0.093

Table S2. Differentially abundant bacterial genera in post-COVID participants following probiotic intervention compared with control. Log-fold change (LFC) coefficients were estimated from paired (t1-t0) changes in genus-level abundance using linear modeling. . p < 0.05 was considered statistically significant, and p < 0.10 was considered a trend. SE, standard error.

Genus	LFC Coefficient	SE	p value
Adlercreutzia	3.80	1.41	0.013
***Ruminococcaceae* DTU089**	2.43	0.94	0.017
Negativibacillus	3.01	1.23	0.024
Eubacterium xylanophilum group	3.07	1.26	0.024
**Unclassified Tannerellaceae**	1.68	0.71	0.028
Holdemanella	1.48	0.72	0.052
Coprococcus	2.96	1.50	0.062
***Eubacterium coprostanoligenes* group**	2.06	1.10	0.075
Anaerostipes	2.19	1.22	0.087
***Eubacterium hallii* group**	1.45	0.81	0.087
***Erysipelotrichaceae* UCG-003**	2.28	1.29	0.092
Gordonibacter	1.22	0.70	0.094
Monoglobus	2.04	1.18	0.099
Oscillospira	1.62	0.96	0.108
Collinsella	1.89	1.13	0.109

Table S3. MetaCyc metabolic pathways showing trend-level enrichment following probiotic supplementation in overall cohort. Functional pathway abundances were predicted from 16S rRNA gene data using PICRUSt2 and summarized at the MetaCyc level. Effect estimates were obtained using linear model analysis to evaluate changes in pathway abundance between probiotic and control groups over time.

**Pathway ID**	**MetaCyc pathways**	**Effect estimate**	**SE**	**T-statistic**	***p* value**
**P281-PWY**	3-phenylpropanoate degradation	0.337	0.175	1.926	0.058
**PWY-6892**	Thiamine diphosphate biosynthesis I	0.093	0.053	1.753	0.084
**PANTO-PWY**	Pantothenate (vitamin B5) biosynthesis	0.095	0.055	1.722	0.089
**TRPSYN-PWY**	L-tryptophan biosynthesis	0.098	0.057	1.721	0.090
**PWY-6897**	Thiamin salvage II	0.091	0.053	1.713	0.091
**PWY-5695**	Urate biosynthesis/inosine 5'-phosphate degradation	0.097	0.057	1.712	0.091
**PWY-6612**	Superpathway of tetrahydrofolate biosynthesis	0.099	0.058	1.689	0.096
**FOLSYN-PWY**	Tetrahydrofolate (folate) biosynthesis	0.092	0.055	1.672	0.099

Table S4. Spearman correlation analysis results for all participants showing significant associations between changes (Δ, t1-t0) in CLR-transformed genus abundances and biochemical markers of liver function (ALP, ALT, AST), CK, CRP, D dimer, ferritin and LDL. Only correlations with |ρ| > 0.3 and p < 0.05 for selected COVIDbiochemical markers are shown.

Genus	Biomarker	ϱ	p value
***Coriobacteriaceae* UCG-002**	ALP	0.379	0.001
Lachnoclostridium	ALT	-0.365	0.002
Oscillibacter		-0.352	0.002
***Lachnospiraceae* UC5-1-2E3**	AST	0.300	0.010
Collinsella	CK	-0.335	0.005
Dorea		-0.324	0.006
Christensenella		-0.310	0.009
Subdoligranulum		-0.308	0.009
Eisenbergiella		-0.303	0.011
Peptoniphilus	CRP	0.325	0.005
**Unclassified Anaerovoracaceae**	D_dimer	-0.340	0.005
Cryptobacterium	Ferritin	-0.338	0.004
Oscillospiraceae		-0.306	0.009
Agathobacter	LDL	-0.349	0.003

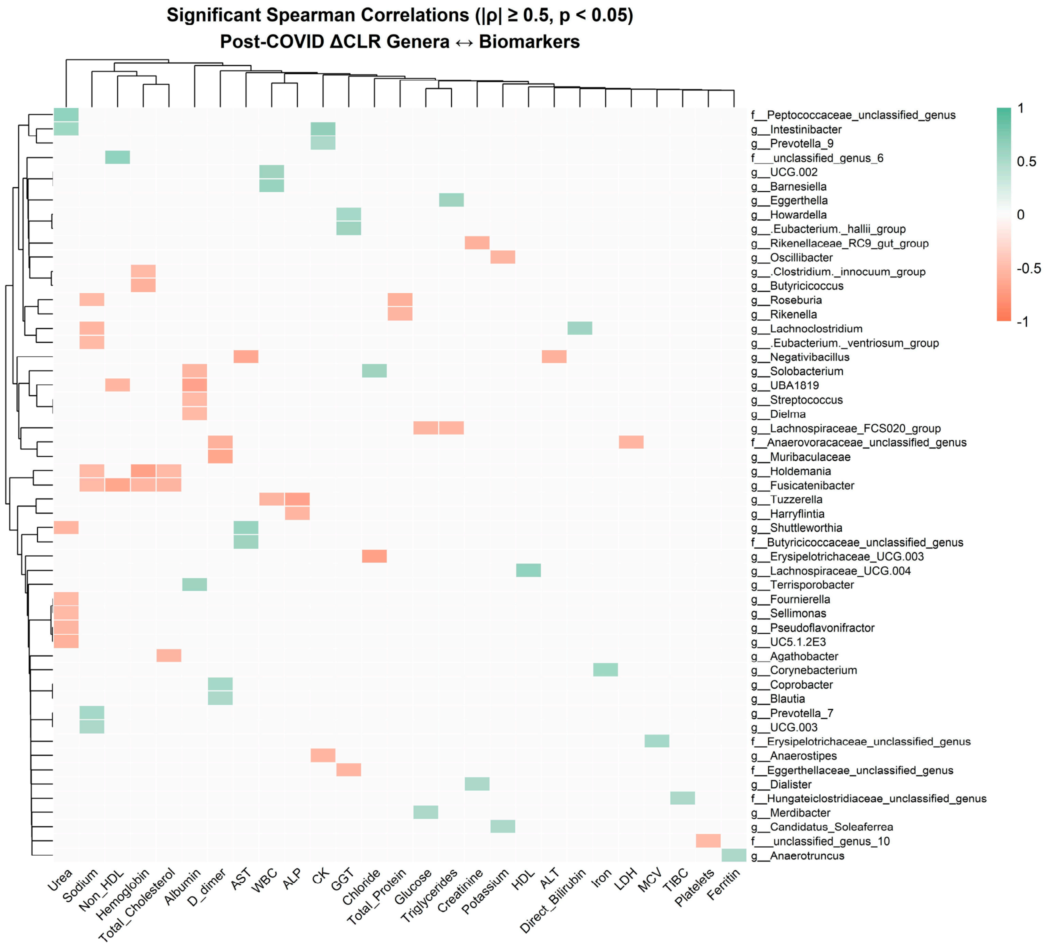

Figure S1. Clustered heatmap of Spearman correlation displaying Spearman correlation coefficients (ρ) between changes in CLR-transformed gut microbial genera and biochemical parameters in long COVID individuals. ρ > 0.5, p < 0.05; Green indicated positive correlations and red indicate negative correlations

Table S5. Spearman correlation analysis results for long COVID affected individuals showing significant associations between changes (Δ, t1-t0) in CLR-transformed genus abundances and biochemical markers of liver function (ALT, AST), systemic inflammation (CRP), D-dimer, ferritin, HDL, and non-HDL. Selected correlations with |ρ| > 0.3 and p < 0.05 for biochemical markers are shown.

Genus	Biomarker	ϱ	p value
Negativibacillus		-0.627	0.002
Slackia		0.505	0.017
Eisenbergiella		0.488	0.021
Shuttleworthia	ALT	0.484	0.023
Lachnospiraceae UC5-1-2E3		0.452	0.035
Eubacterium brachy group		-0.434	0.043
Prevotellaceae NK3B31 group		0.427	0.047
Negativibacillus		-0.731	0.0001
Eubacterium coprostanoligenes group		-0.511	0.015
Actinomyces	AST	-0.496	0.019
Oscillospiraceae UCG-002		-0.483	0.023
Collinsella		-0.434	0.044
Oscillospiraceae UCG-005		0.487	0.021
Frisingicoccus		-0.476	0.025
Tannerellaceae	CRP	-0.459	0.032
Gordonibacter		-0.443	0.039
Holdemanella		-0.427	0.047
Intestinibacter		0.568	0.007
Prevotella_9		0.562	0.008
Collinsella		-0.525	0.015
CAG-56		0.517	0.016
Barnesiella		-0.513	0.017
Anaerofilum	CK	0.506	0.019
Clostridium_sensu_stricto_1		0.480	0.028
Coprobacter		-0.466	0.033
Anaerostipes		-0.448	0.042
Erysipelotrichaceae UCG-003		-0.436	0.048
Blautia		0.650	0.003
Collinsella	D_dimer	0.536	0.018
Sellimonas		0.460	0.047
Lachnospiraceae UCG-004		0.682	0.000
Unclassified Lachnospiraceae		-0.522	0.013
UBA1819		-0.510	0.015
UC5.1.2E3		-0.483	0.023
Eggerthella	HDL	-0.466	0.029
Ruminococcus_gnavus_group		-0.453	0.034
Holdemania		-0.436	0.043
Lachnospiraceae ND3007 group		0.433	0.044
Fusicatenibacter		-0.675	0.001
Unclassified_6	Non_HDL	0.662	0.001
Eubacterium brachy group		-0.522	0.018
Senegalimassilia		0.510	0.022

UBA1819	-0.491	0.028
Terrisporobacter	0.467	0.038
